# An upgraded *Myxococcus xanthus* chassis with enhanced growth characteristics for efficient genetic manipulation

**DOI:** 10.1016/j.engmic.2024.100155

**Published:** 2024-06-03

**Authors:** Weifeng Hu, Yan Wang, Xiaoran Yue, Weiwei Xue, Wei Hu, Xinjing Yue, Yuezhong Li

**Affiliations:** State Key Laboratory of Microbial Technology, Institute of Microbial Technology, Shandong University, Qingdao 266237, China

**Keywords:** *Myxococcus xanthus*, Operation deficiency, Phenotypic optimization, Chassis, Genetic performance

## Abstract

Myxobacteria are well known for multicellular social behaviors and valued for biosynthesis of natural products. Myxobacteria social behaviors such as clumping growth severely hamper strain cultivation and genetic manipulation. Using *Myxococcus xanthus* DK1622, we engineered Hu04, which is deficient in multicellular behavior and pigmentation. Hu04, while maintaining nutritional growth and a similar metabolic background, exhibits improved dispersed growth, streamlining operational procedures. It achieves high cell densities in culture and is promising for synthetic biology applications.

## Graphical abstract

**Figure:**
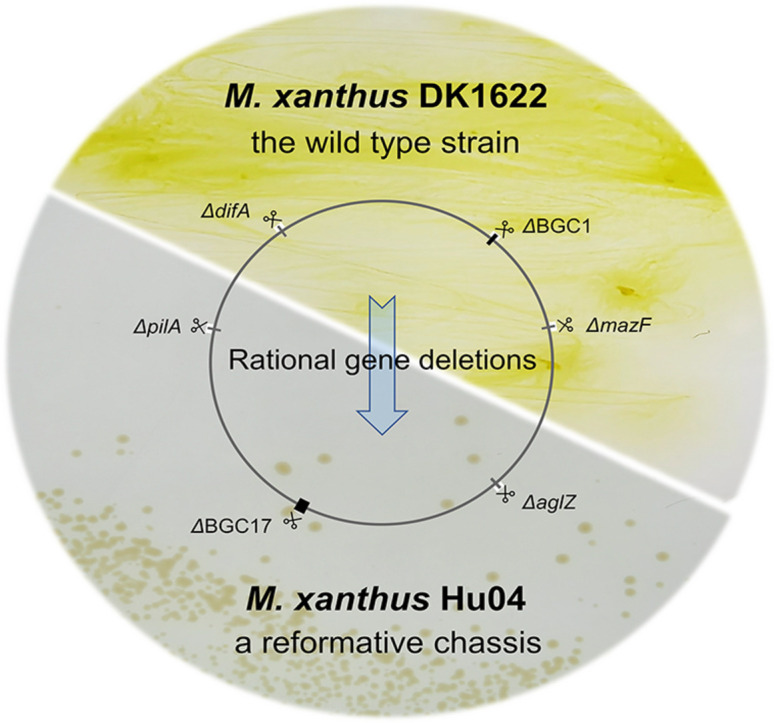

Myxobacteria are increasingly recognized as a significant reservoir of natural products (NPs), having provided over 100 unique core structures and approximately 500 derivatives [1,2]. Due to limitations of slow growth and difficulties in genetic manipulation of most myxobacteria, biosynthetic gene clusters (BGCs) identified through genome mining and required for NP production often have to be heterologously expressed in a myxobacteria model strain, *Myxococcus xanthus* DK1622 [3]. An ideal expression chassis is expected to offer optimized growth characteristics, efficient genetic manipulation, and adequate precursors [[4], [5], [6]]. Although widely used as the only chassis for myxobacteria engineering, wild-type DK1622 is still inadequate for laboratory manipulations because it requires cumbersome cultivation and genetic manipulations, and is subject to cell aggregation, adhesion, phase variation, and slow growth, which significantly disrupts routine operations and may complicate interpretation of results [3]. For instance, *M. xanthus* cells must be incubated in flasks, rather than in handy Eppendorf (EP) tubes, for liquid culture due to cellular clumping during growth, and purification of a traceless-knockout mutant typically requires more than one month due to high-frequency heterozygous colonies. Such difficulties emphasize the necessity and urgency of optimizing *M. xanthus* as a chassis for synthetic biology research and for the exploitation of untapped NPs in myxobacteria.

The fascinating multicellular social characteristics of myxobacteria, including its dual motility system, aggregation, and fruiting body development [7], are key factors hindering efficient genetic manipulation and strain cultivation. The well-explored genetic background of *M. xanthus* DK1622 offers the possibility of rational design engineering to improve cell traits for ease of laboratory manipulation. For example, *aglZ* which encodes the filamentous curlin protein, is a pivotal gene for adventurous (A) motility [8], whereas the *pilA*, which encodes the PilA protein for the formation of type IV pili, is required for social (S) motility [9]. Cell adhesion in myxobacteria is intricately linked to the presence of substantial extracellular polysaccharides (EPS), and *pilA* and *difA* play critical roles in the regulation of EPS biosynthesis [9,10]. Of the 24 endogenous BGCs in the DK1622 genome, BGC1 and BGC17 are responsible for the synthesis of colored carotenoids [11] and Dkxanthene [12], respectively. The synthesis of red carotenoids is light-regulated, and inadequate control of culture conditions may result in color variation. Dkxanthene is required for developmental sporulation [13] and instability in pigment molecule production may be associated with phase variation, resulting in significant color fluctuations that interfere with observations and assessments. Moreover, the *mazF* gene is involved in programmed cell death in *M. xanthus*, and its deletion eliminates programmed cell death during development [14].

In the present study, we systematically deleted the aforementioned genes or genetic regions to selectively improve DK1622 growth and genetic performance to facilitate synthetic biological applications. Detailed gene functions, including *aglZ, pilA, difA*, BGC17 (containing the *dkx* cluster for the production of Dkxanthene), and BGC1 (the *car* cluster for carotenoid synthesis) are documented in Supplementary Fig. S1. Approximately 95-Kb chromosomal DNA fragments containing more than 50 genes (Supplementary Table S1) were removed, and the mutant strain was named *M. xanthus* Hu04. Target region knockouts were verified by genome resequencing. When compared with the DK1622 genome sequence in GenBank (accession number: NC_008095.1), some unexpected mutations, predominantly consisting of single nucleotide polymorphisms (SNPs), were found in the Hu04 genome (Supplementary Table S2). These non-synonymous mutations impact one transfer RNA (tRNA-Ile) and 29 coding genes, including genes encoding transcriptional regulators, transporters, and non-ribosomal peptide synthetases. However, none of them are associated with social behaviors, according to a summary of previous studies [15]. We speculate that these unexpected mutations may have originated as spontaneous mutations during the year-long cultivation and serial passaging.

With the selected social behavior genes deleted, Hu04 was phenotypically distinct from its parental strain (Fig. 1). Compared with DK1622 cells, Hu04 cells showed defective S-motility (Fig. 1A, left). With decreased A-motility outside of a colony, the expansion-growing membranes of Hu04 colonies narrowed significantly (Fig. 1A, right). When incubated in a nutrient-limited medium (TPM), Hu04 completely lost its aggregation ability and failed to form fruiting bodies (Fig. 1B). Due to BGC17 and BGC1 knockouts, the colony color changed from bright yellow in DK1622 to light yellow in Hu04; Light treatment induced a yellow-red color transformation in DK1622 but not in Hu04 (Fig. 1C). Therefore, deletion of the corresponding genes successfully perturbed multicellular behavior and pigment synthesis in Hu04 cells.

**Fig. 1:**
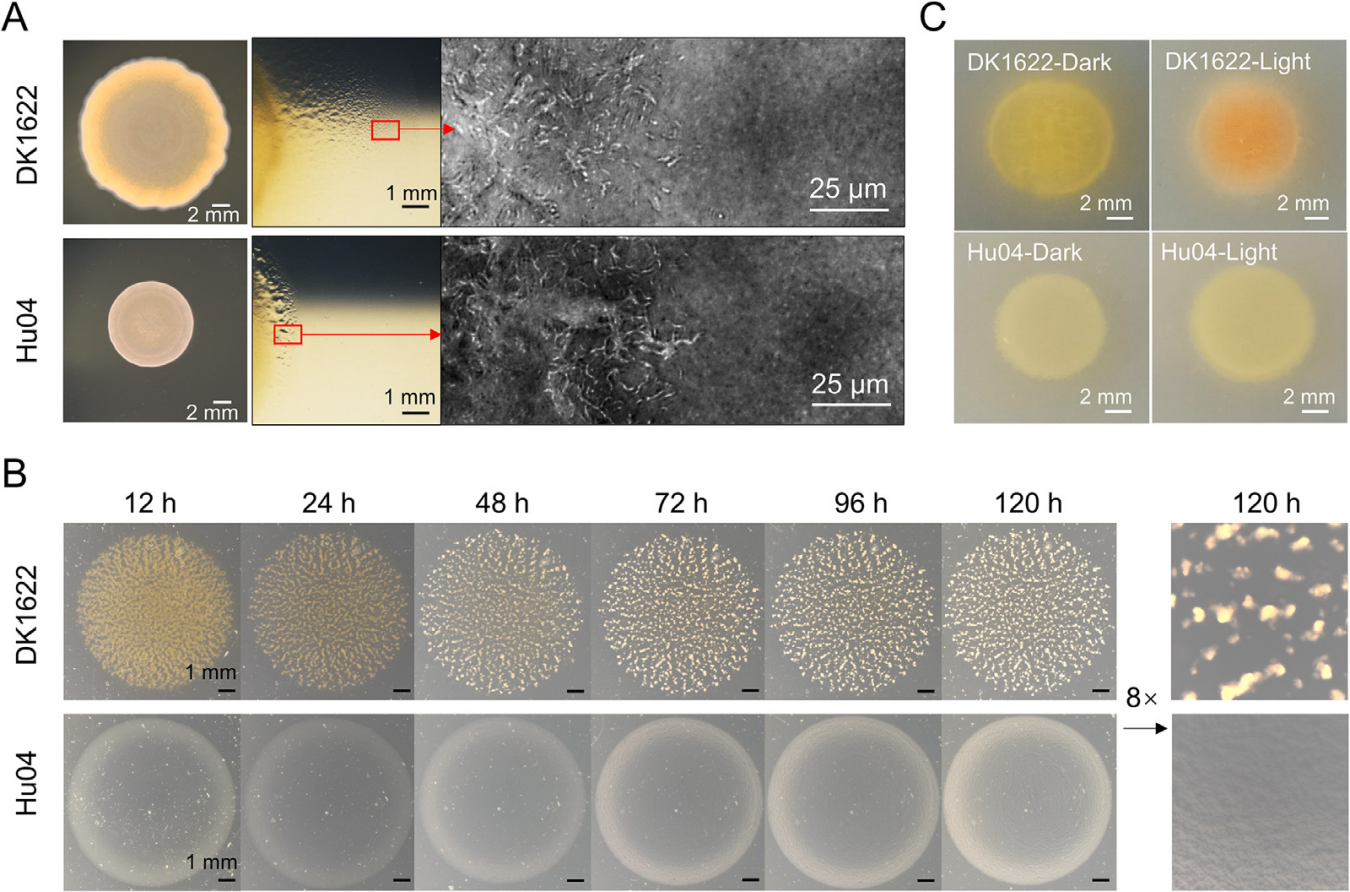
Comparison of social behaviors and pigment synthesis in *M. xanthus* DK1622 and Hu04. (A) Social motility (left; on soft CTT plate containing 0.3 % agar) and adventurous motility (right; on hard CTT plate containing 1.5 % agar) of DK1622 and Hu04. (B) DK1622 and Hu04 fruiting body formation. **(C)** DK1622 and Hu04 colony colors.

We further evaluated the growth and cellular characteristics of *M. xanthus* Hu04 cells. Under normal cultivation in flasks, Hu04 exhibited a growth curve similar to that of DK1622, with a slight reduction in maximum biomass in the stationary phase (Fig. 2A). Scanning electron microscopy revealed that Hu04 cell size and morphology were almost unchanged, but fewer vesicle-like protrusions [16] were found on the surface of Hu04 cells (Fig. 2B). Notably, the Hu04 strain did not adhere to flask walls (Fig. 2C). Unlike wild-type DK1622, the mutant Hu04 exhibited better cultivation performance from inoculating a single clone into a 10 mL centrifuge tube or a 1.5-ml EP tube, without flocculation (Fig. 2D), which will greatly facilitate genetic manipulation, especially large-scale strain screening procedures. Under high-density fermentation conditions, Hu04 demonstrated superior traits compared with DK1622. The mutant exhibited increased biomass and an extended plateau phase, with corresponding changes in dissolved oxygen and pH in the fermenter (Fig. 2E). Moreover, Hu04 maintained a uniform cell distribution throughout fermentation, avoiding vessel adhesion and the formation of large cell aggregates or biofilms (Fig. 2F). Additionally, despite dramatic improvements in cultivation phenotypes, High-Performance Liquid Chromatography (HPLC) analysis showed that Hu04 did not possess a significantly altered metabolic background (Fig. 2G), indicating the preservation of its metabolic network.

**Fig. 2:**
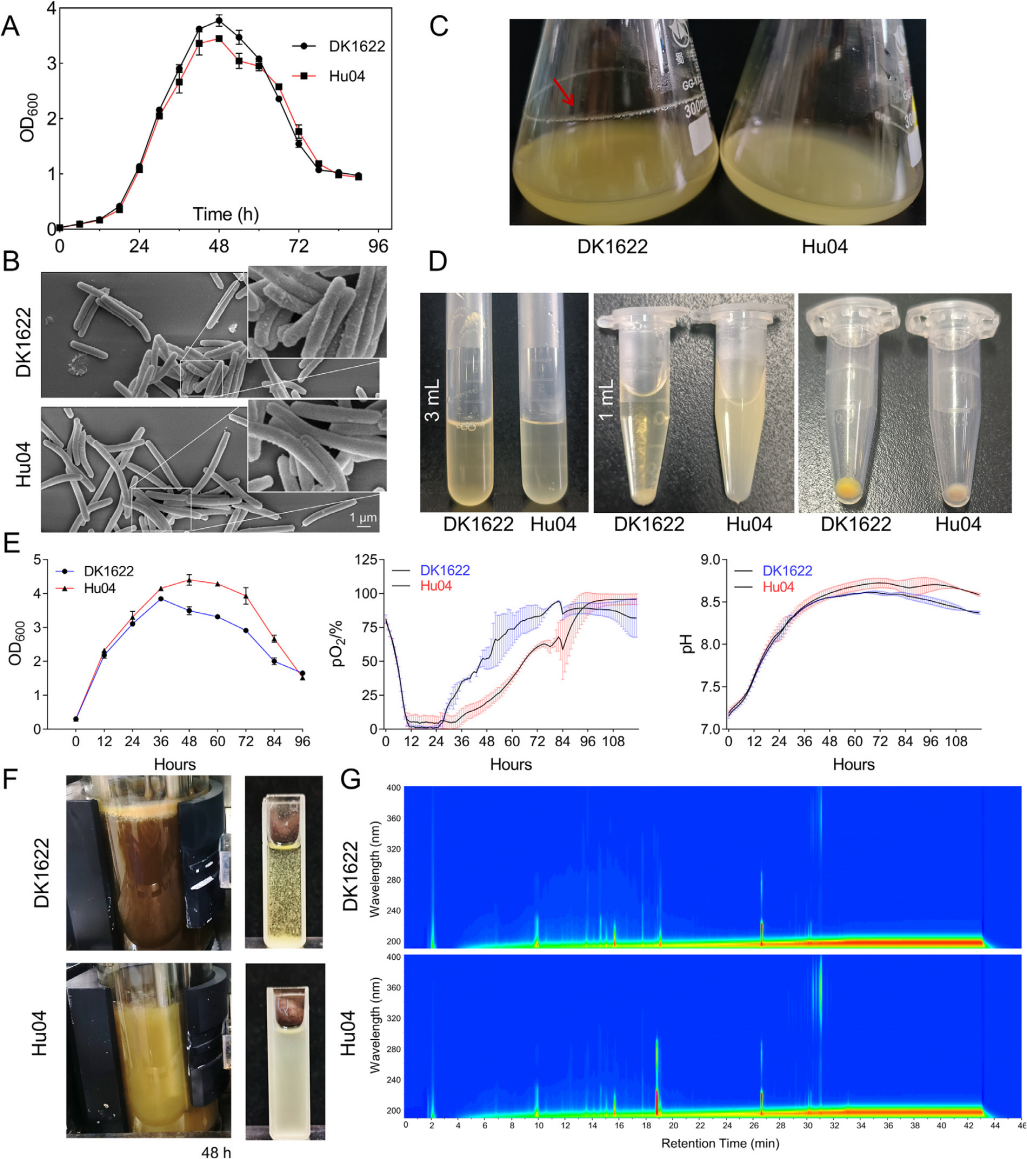
Growth characteristics of *M. xanthus* Hu04. (A) DK1622 and Hu04 growth curves. (B) DK1622 and Hu04 cell morphology. (C) DK1622 and Hu04 incubated in flasks. DK1622 cells adherent to flask walls are marked with a red arrow. (D) DK1622 and Hu04 cultivated in centrifuge tubes and EP tubes. Left: DK1622 and Hu04 cultured in 10 mL centrifuge tubes for 2 days; Middle: DK1622 and Hu04 cultured in 1.5 mL-EP tubes for 2 days; Right: cultivated DK1622 and Hu04 cells. (E) Cell growth, dissolved oxygen levels, and pH variations during high-density fermentation. Fermentation is conducted using a parallel bioreactor with a 1 L volume. (F) Fermentation characteristics at 48 h. A three-fold diluted bacterial solution is displayed on the right. (G) Metabolite profile comparison. Fermentation products adsorbed on XAD-16 resin were extracted with methanol. Scanning wavelengths for HPLC ranged from 190 to 400 nm. the *x*-axis displays HPLC time (min) and the *y*-axis represents absorption wavelength (nm).

We expressed different proteins in Hu04 to demonstrate its synthetic capabilities. With the introduction of the gene encoding the green fluorescent protein (eGFP) under the control of a strong promoter, bands of eGFP, approximately 27 kDa, were detected in the total protein extracted from disrupted bacterial cells (Fig. 3A and B), confirming similar protein expression in DK1622 and Hu04. The endogenous Type I PKS enzyme MchA (∼230 kDa), catalyzing the first step in the silent *mch* BGC for the production of orange-colored myxochromide [17], was overexpressed under the control of the isopropyl-beta-d-thiogalactopyranoside (IPTG)-inducible promoter in DK1622 and Hu04. IPTG induction of MchA led to a noticeable color change in both DK1622 and Hu04, indicating accumulation of the pigment compound (Fig. 3C and Supplementary Fig. 2A), and the induced MchA protein was easily observed in a total protein sample (Fig. 3D). Subsequently, using a 6×His-tag at the N-terminus of MchA, we successfully purified recombinant MchA protein from Hu04, marking the purification of the first native myxobacterial PKS enzyme. The expression of eGFP and MchA illustrates the potential of Hu04 cells as a chassis for expressing proteins and NPs for further synthetic biology applications.

**Fig. 3:**
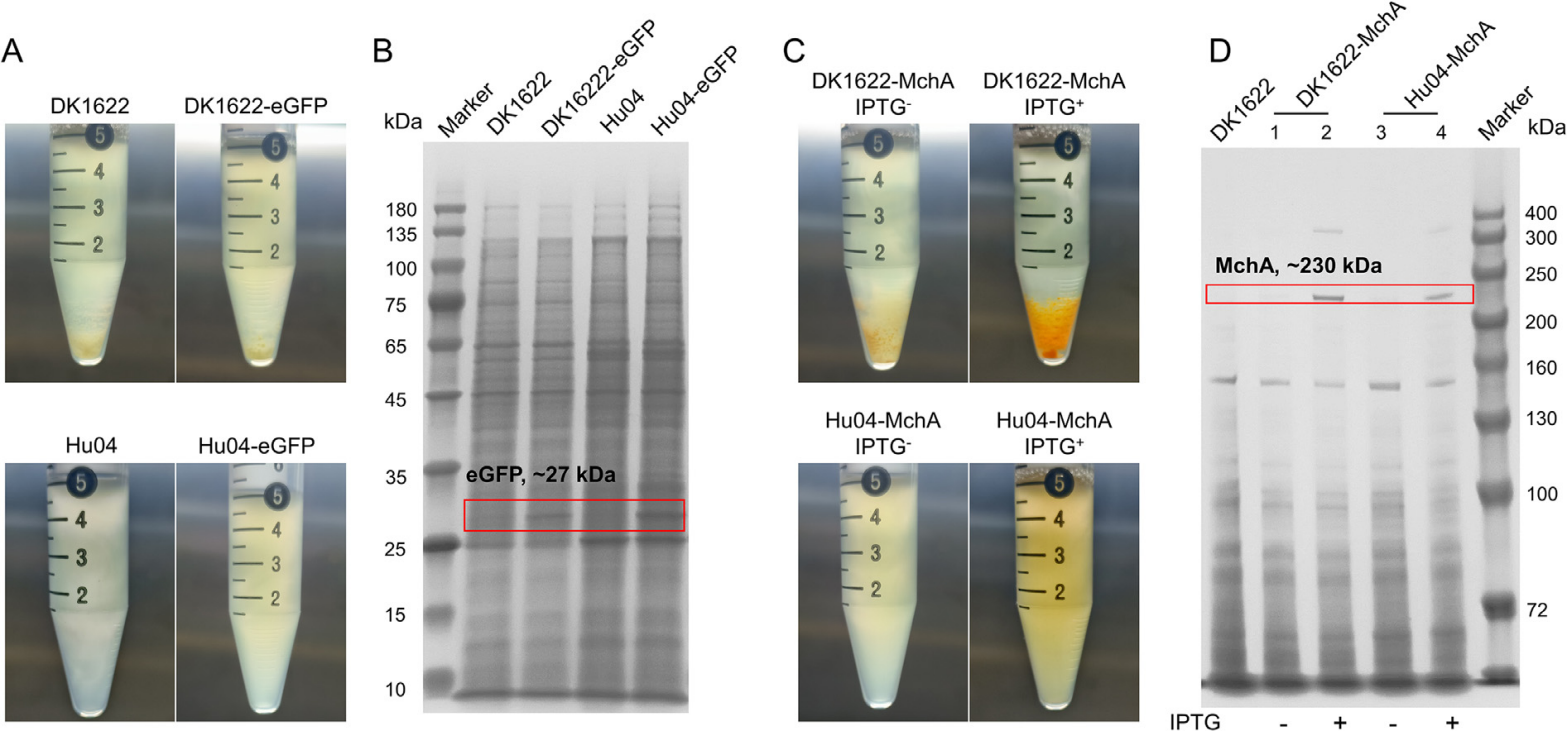
Comparison of DK1622 and Hu04 protein and secondary metabolite expression. (A) Expression of the fluorescent protein eGFP. DK1622-eGFP: DK1622 transformed with an eGFP expression vector; Hu04-eGFP: Hu04 transformed with an eGFP expression vector. (B) Bands of eGFP appear in the SDS page of total protein. The approximately 27 kDa band (eGFP) was not observed in control cultures lacking eGFP. (C) Expression of the starting PKS enzyme MchA. The induction of MchA by IPTG activated the secondary gene cluster *mch*, resulting in the production of orange-colored compounds. (D) Bands of MchA were observed in the SDS-page analysis of total protein. The approximately 230 kDa band of MchA appeared following the addition of IPTG.

With Hu04, an improved procedure for cultivation and genetic manipulation was finally constructed. Taking the example of electroporating a vector carrying eGFP (Fig. 4), the application of frozen competent cells, rather than *de novo* activated cells, simplified preprocessing for electroporation. After electroporation, the resuspended bacterial solution was spread directly on screening plates without pre-mixing with soft agar. This approach may help reduce the occurrence of false-positive colonies, heterozygous colonies, and potential inaccuracies in the statistical analysis of mutations. Mutant clones were picked into EP tubes for small-scale cultivation, omitting the strain-transfer step to new plates. In summary, compared with the traditional procedure for DK1622 (Fig. 4A), the modified manipulation procedure for Hu04 (Fig. 4B) can save at least one week in the construction of each new *M. xanthus* mutant.

**Fig. 4:**
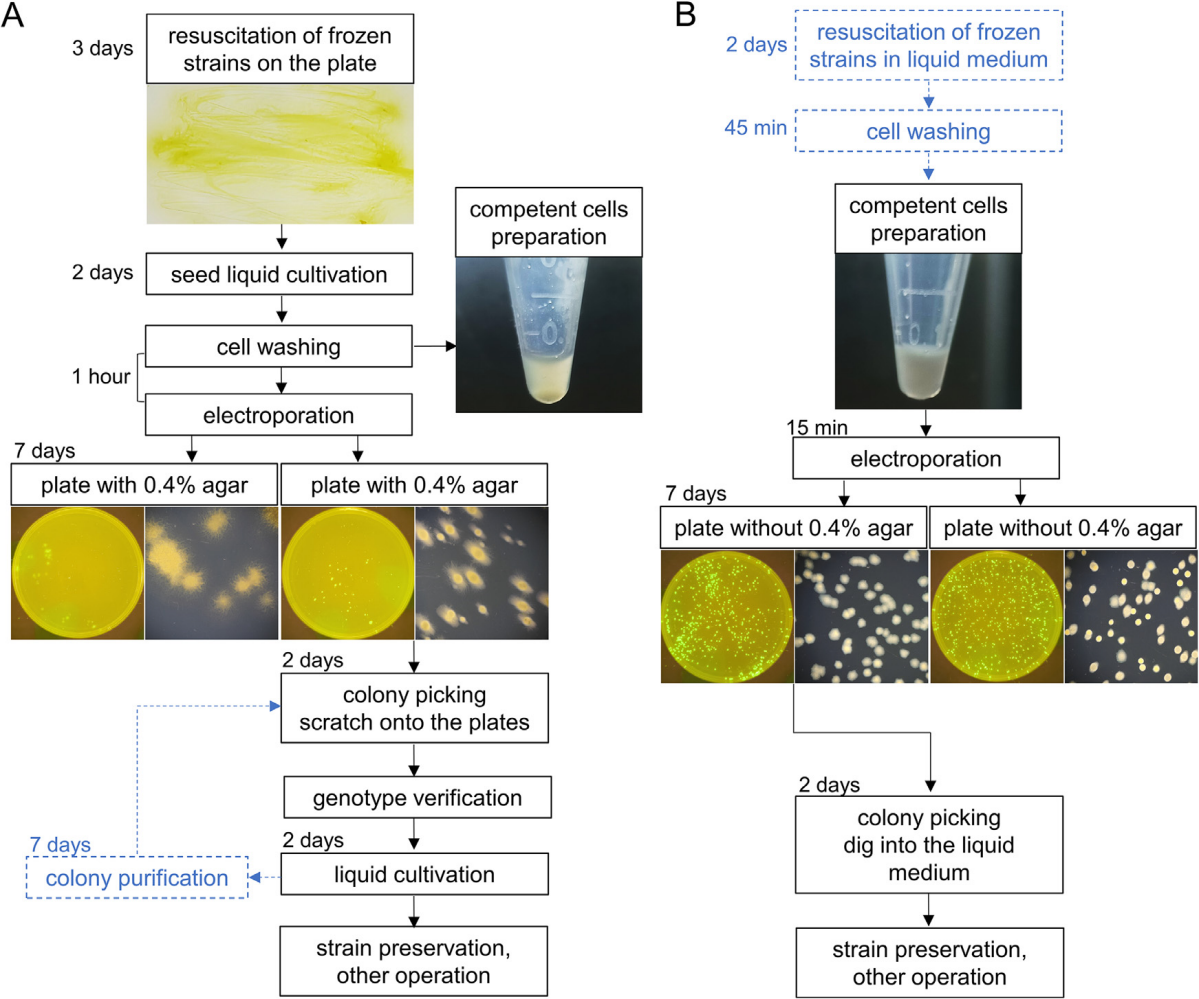
Electroporation procedures for DK1622 (A) and Hu04 (B). DK1622 was revived on agar plates, while Hu04 was directly cultured in a liquid medium. Prolonged stasis during the thawing of DK1622 resulted in cellular aggregation and settling, necessitating cell washing before each electroporation. Cells transformed with the pZJY41-eGFP vector were subjected to electroporation and subsequently plated in 0.4 % soft agar or directly spread. Colony morphologies were photographed under blue light using a camera, and magnified images were captured using a stereomicroscope. The blue box outline indicates that this step was not performed in every procedure.

In conclusion, by rational engineering of *M. xanthus* DK1622, we obtained the laboratory-friendly strain chassis Hu04 with defective multicellular behaviors, but enhanced convenience and efficiency in cultivation and genetic manipulation. This upgraded chassis strain is intended to address research needs related to myxobacterial synthetic biology: (1) expressing/purifying proteins, (2) characterizing host heterologous gene clusters, (3) screening genetic parts, (4) constructing engineered bacteria, and (5) simplifying the genome. The genome design strategies adopted in this paper, as well as in other studies, could effectively support synthetic biology and industrial biotechnology [[18], [19], [20]].

## Data availability

All data generated or analyzed during this study are included in this published article and its supplementary information files or are available upon request.

## CRediT authorship contribution statement

**Weifeng Hu:** Writing – original draft, Visualization, Investigation, Formal analysis, Data curation, Conceptualization. **Yan Wang:** Visualization, Validation, Formal analysis, Data curation. **Xiaoran Yue:** Methodology, Data curation. **Weiwei Xue:** Methodology, Data curation. **Wei Hu:** Validation, Formal analysis. **Xinjing Yue:** Writing – review & editing, Validation, Supervision, Funding acquisition. **Yuezhong Li:** Writing – review & editing, Supervision, Funding acquisition, Conceptualization.

## Declaration of Competing Interest

Given his role as editorial board member, Dr. Yuezhong Li, had no involvement in the peer-review of this article and has no access to information regarding its peer-review. Full responsibility for the editorial process for this article was delegated to Dr. Shengbiao Hu.

## Appendix Supplementary materials

Image, application 1
