# Supplementary material for: An upgraded Myxococcus xanthus chassis with enhanced growth characteristics for efficient genetic manipulation
Source: Eng Microbiol. 2024 Jun 3;4(3):100155. doi: 10.1016/j.engmic.2024.100155 (PMC11611028; doi:10.1016/j.engmic.2024.100155)
Supplement: Supplementary file 1 [file mmc1.docx]

**Supporting Information for：**

**An upgraded *Myxococcus xanthus* chassis with enhanced growth characteristics for efficient genetic manipulation**

Wei-feng Hu^1^, Yan Wang^1^, Xiao-ran Yue^1^, Wei-wei Xue^1^, Wei Hu^1^, Xin-jing Yue^1*^, Yue-Zhong Li^1*^

^1^State Key Laboratory of Microbial Technology, Institute of Microbial Technology, Shandong University, 266237 Qingdao, P.R. China

*** The corresponding authors:** Xin-jing Yue, xjy2018@sdu.edu.cn; ORCID ID: 0000-0002-0423-3712; Yue-zhong Li, lilab@sdu.edu.cn; ORCID ID, 0000-0001-8336-6638.

Materials and methods

**1. Strains and cultural conditions**

*Myxococcus xanthus* strains were cultivated in CTT medium (10g/L casitone, 10 mM Tris-HCl, 1 mM K_2_HPO_4_/KH_2_PO_4_, 8 mM MgSO_4_, pH 7.6) with 200 rpm shaking at 30 °C. LB medium was used to culture *Escherichia coli* strains at 37 °C. Solid agar plates were prepared by adding 1.5% agar. TPM plate (10 mM Tris-HCl, 1 mM K_2_HPO_4_/KH_2_PO_4_, 8 mM MgSO_4_, pH 7.6, 1.5% agar) was used to observe the formation of the fruiting body. When needed, the kanamycin (Km, 40 μg/mL) or apramycin (Apra, 60 μg/mL) was added to screen the transformants. The strains used in this study are listed in Supplementary Table S3.

**2. Plasmid construction**

All plasmids were constructed using the ClonExpress Ultra One Step Cloning Kit V2 C116 (Vazyme, Nanjing, PRC). With pBJ113 plasmid as templet, the linearized backbone of knockout plasmid was amplified with primer pair 113-F/113-R. Homologous upstream and downstream (~1 kb respecctively) of the target regions were amplified from DK1622 genome and then annealed with the linearized pBJ113 backbone to construct the knockout plasmids. With the same procedure, the eGFP governed by the promoter pSH058 [1] were amplified from pBJ113-eGF*P*, respectively, and then cloned into the pZJY41 plasmid, resulting in the fluorescent reporter plasmids pZJY41-eGFP. In the case of the MchA expression vector, multiple amplified fragments were cloned from the plasmids pZJY41, pBJ113-Lachis, and pTim-S0 (The features of the plasmids were listed in Supplementary Figure S3), and then simultaneously recombined together. Primer details are shown in Supplementary Table S4.

**3. Construction of the *M. xanthus* mutants**

Gene deletion was achieved through classical homologous recombination as previously described [2]. The expression vectors of fluorescins and *mchA* were introduced into *M. xanthus* Hu04 by electroporation, and the mutants were screened with Km or Apra, respectively. Subsequently, genotype verification was performed with colony PCR and sequencing.

**4. Genome re-sequencing**

*M. xanthus* Hu04 was cultured in CTT medium and harvested at the exponential phase. The genomic DNA was extracted and then fragmented to a size of 350 bp by sonication. Following ligation with the full-length adapter and PCR amplification, the qualified libraries were pooled and subjected to sequencing on Illumina platforms using a PE150 strategy at Allwegene Company (Beijing, China). The Sequence Read Archive (SRA) data of the re-sequencing was uploaded to the NCBI public database with the BioProject ID: PRJNA1057470

**5. Multicellular social behaviors assay**

*M. xanthus* Hu04 cells were collected at exponential stage, washed with TPM buffer three times, and then resuspended to approximately 5×10^9 cells/mL. 2 μL of suspension was inoculated onto semi-solid CTT plates containing 0.3% agar, and incubated at 30 °C in the dark for 3-4 days. The social motility was characterized according to the colony diameter [3]. 5 μL of suspension was incubated on CTT plates at 30 °C in the dark for 3-4 days, the colony color and adventurous motility were observed visually and with the microscope, respectively. The colonies cultured under light were observed for comparison. 10 μL of suspension incubated on TPM plates was observed periodically with a stereomicroscope to record cell aggregation and fruiting body formation.

**6. Preparation of competent cells**

*M. xanthus* was cultivated in 50 mL of CTT liquid medium overnight, and harvested at exponential phase by centrifugation at 7,585 × g for 5 minutes. The cells were washed with 10% glycerol buffer three times, resuspended in 2.5 mL of 10% glycerol, subpackaged as 50 μL per tube, and then stored at -80 °C.

**7. Modified electroporation procedure for *M. xanthus* Hu04**

The competent cells were pre-thawed on ice, mixed with the plasmid, and transferred into a 2-mm cuvette to perform electroporation under the conditions 625 V/mm, 400 Ω, 25 μF. Subsequently, 1 mL of CTT medium was added to rinse cells, which were then incubated in 1.5 ml EP tubes with perforated lids for 2 hours. After that, the appropriate antibiotic was supplemented for continued incubation for 2 hours. The culture was then spread on the screening plates, and the single clone appearing after 7 days of incubation was picked for genotype testing. No further purification of the mutant is required.

**8. Expression and purification of proteins in *M. xanthus***

The mutant *M. xanthus* introduced with expression vector was inoculated in 50 mL of CTT medium until the stationary phase. 10 mL of the culture was transferred into 500 mL of fresh medium, and cultivated at 30 °C for 24 hours, followed by induction with IPTG for 36 hours. Induced cells were collected and resuspended in a 10% volume of Lysis Buffer (20 mM Tris, 250 mM NaCl, 20 mM imidazole, 10% glycerol, pH=8.0), and then disrupted using a high-pressure homogenizer. After centrifugation at 14,339 × g, 4 °C for 30 minutes, the protein supernatant was filtered with a 0.8-μm membrane and further purified by Ni-affinity chromatography with AKTA Avant FPLC. Purified proteins were analyzed by SDS-PAGE.

**Supplementary Table S1.** Information of knockout genes and regions.

| KO Region | Start | End | Length (nt) | Gene ID |
| --- | --- | --- | --- | --- |
| Δ*difA* | 8,235,892 | 8,237,121 | 1230 | MXAN_RS32420 |
| Δ*pilA* | 7,158,853 | 7,159,411 | 559 | MXAN_RS28035 |
| Δ*aglZ* | 3,505,100 | 3,507,752 | 2653 | MXAN_RS14485 |
| ΔBGC17 | 5,235,902 | 5,311,072 | 75171 | MXAN_RS20740 |
|  |  |  |  | MXAN_RS20745 |
|  |  |  |  | MXAN_RS20750 |
|  |  |  |  | MXAN_RS20755 |
|  |  |  |  | MXAN_RS20760 |
|  |  |  |  | MXAN_RS20765 |
|  |  |  |  | MXAN_RS20770 |
|  |  |  |  | MXAN_RS20775 |
|  |  |  |  | MXAN_RS20780 |
|  |  |  |  | MXAN_RS20785 |
|  |  |  |  | MXAN_RS20790 |
|  |  |  |  | MXAN_RS20795 |
|  |  |  |  | MXAN_RS20800 |
|  |  |  |  | MXAN_RS38200 |
|  |  |  |  | MXAN_RS20805 |
|  |  |  |  | MXAN_RS20810 |
|  |  |  |  | MXAN_RS20815 |
|  |  |  |  | MXAN_RS20820 |
|  |  |  |  | MXAN_RS20825 |
|  |  |  |  | MXAN_RS20830 |
|  |  |  |  | MXAN_RS20835 |
|  |  |  |  | MXAN_RS20840 |
|  |  |  |  | MXAN_RS20845 |
|  |  |  |  | MXAN_RS20850 |
|  |  |  |  | MXAN_RS20855 |
|  |  |  |  | MXAN_RS20860 |
|  |  |  |  | MXAN_RS20865 |
|  |  |  |  | MXAN_RS20870 |
|  |  |  |  | MXAN_RS20875 |
|  |  |  |  | MXAN_RS20880 |
|  |  |  |  | MXAN_RS20885 |
|  |  |  |  | MXAN_RS20890 |
|  |  |  |  | MXAN_RS20895 |
|  |  |  |  | MXAN_RS20900 |
|  |  |  |  | MXAN_RS20905 |
|  |  |  |  | MXAN_RS20910 |
| ΔBGC1 | 1,015,682 | 1,031,122 | 15441 | MXAN_RS04270 |
|  |  |  |  | MXAN_RS04275 |
|  |  |  |  | MXAN_RS04280 |
|  |  |  |  | MXAN_RS04285 |
|  |  |  |  | MXAN_RS04290 |
|  |  |  |  | MXAN_RS04295 |
|  |  |  |  | MXAN_RS04300 |
|  |  |  |  | MXAN_RS04305 |
|  |  |  |  | MXAN_RS04310 |
|  |  |  |  | MXAN_RS04315 |
|  |  |  |  | MXAN_RS04320 |
|  |  |  |  | MXAN_RS04325 |
|  |  |  |  | MXAN_RS04330 |
|  |  |  |  | MXAN_RS04335 |
|  |  |  |  | MXAN_RS04340 |
|  |  |  |  | MXAN_RS04345 |
| Δ*mazF* | 1,962,661 | 1,963,028 | 368 | MXAN_RS08055 |

**Supplementary Table S2.** Variations and mutations in the *M. xanthus* Hu04 genome.

| **SNPs:** |  |  |  |  |  |  |  |
| --- | --- | --- | --- | --- | --- | --- | --- |
| Chromosome | Position | Reference | Alteration | Quality | Mutation | Locus | Annotation |
| NC_008095.1 | 148110 | C | T | 4160.77 | synonymous | MXAN_RS00630 | CapA family protein |
| NC_008095.1 | 376221 | A | G | 3995.77 | intergenic | - |  |
| NC_008095.1 | 468903 | G | T | 4668.77 | intergenic | - |  |
| NC_008095.1 | 830180 | T | G | 3494.77 | stoploss | MXAN_RS03515 | response regulator transcription factor |
| NC_008095.1 | 1630453 | G | A | 760.77 | synonymous | MXAN_RS06730 | recombinase RecA |
| NC_008095.1 | 1746036 | A | G | 3589.77 | synonymous | MXAN_RS07195 | polysaccharide deacetylase family protein |
| NC_008095.1 | 1752952 | A | G | 3717.77 | unknown | MXAN_RS07240 | tRNA-Ile |
| NC_008095.1 | 1777991 | C | T | 4059.77 | synonymous | MXAN_RS07365 | UvrD-helicase domain-containing protein |
| NC_008095.1 | 1837528 | C | G | 3563.77 | missense | MXAN_RS07605 | sigma 54-interacting transcriptional regulator |
| NC_008095.1 | 2328668 | T | G | 3337.77 | intergenic | - |  |
| NC_008095.1 | 2558460 | C | G | 3436.77 | missense | MXAN_RS10695 | DMT family transporter |
| NC_008095.1 | 2774530 | G | T | 2456.77 | synonymous | MXAN_RS11540 | PAS domain S-box protein |
| NC_008095.1 | 3036248 | C | T | 3610.77 | synonymous | MXAN_RS12605 | peptide MFS transporter |
| NC_008095.1 | 3337759 | G | T | 3847.77 | missense | MXAN_RS13860 | HEAT repeat domain-containing protein |
| NC_008095.1 | 3382602 | A | G | 2907.77 | missense | MXAN_RS37645 | P-loop NTPase fold protein |
| NC_008095.1 | 3396307 | T | G | 3590.77 | intergenic | - |  |
| NC_008095.1 | 4188963 | T | C | 2886.77 | intergenic | - |  |
| NC_008095.1 | 4937257 | C | A | 2836.77 | synonymous | MXAN_RS19595 | carboxypeptidase-like regulatory domain-containing protein |
| NC_008095.1 | 5012519 | C | T | 2377.77 | synonymous | MXAN_RS19835 | non-ribosomal peptide synthetase |
| NC_008095.1 | 5102253 | G | C | 3060.77 | missense | MXAN_RS20170 | HEAT repeat domain-containing protein |
| NC_008095.1 | 5538021 | G | A | 140.9 | synonymous | MXAN_RS21725 | IS3-like element ISMxa1 family transposase |
| NC_008095.1 | 5666970 | C | A | 3450.77 | missense | MXAN_RS22045 | adenylate/guanylate cyclase domain-containing protein |
| NC_008095.1 | 5699584 | C | A | 3096.77 | missense | MXAN_RS22150 | GNAT family N-acetyltransferase |
| NC_008095.1 | 5756401 | G | C | 3674.77 | missense | MXAN_RS22315 | non-ribosomal peptide synthetase |
| NC_008095.1 | 5888001 | T | A | 2819.77 | missense | MXAN_RS22830 | lysine 2,3-aminomutase |
| NC_008095.1 | 5998849 | C | T | 1932.77 | synonymous | MXAN_RS23320 | Ig-like domain-containing protein |
| NC_008095.1 | 5998879 | T | C | 2292.77 | synonymous | MXAN_RS23320 | Ig-like domain-containing protein |
| NC_008095.1 | 5999065 | T | C | 2041.77 | synonymous | MXAN_RS23320 | Ig-like domain-containing protein |
| NC_008095.1 | 6012571 | G | A | 3289.77 | synonymous | MXAN_RS23350 | type VI secretion system membrane subunit TssM |
| NC_008095.1 | 6142311 | G | A | 3852.77 | missense | MXAN_RS23820 | peptide chain release factor N(5)-glutamine methyltransferase |
| NC_008095.1 | 6357202 | G | T | 3094.77 | synonymous | MXAN_RS24700 | replicative DNA helicase |
| NC_008095.1 | 6423302 | G | T | 3310.77 | synonymous | MXAN_RS24985 | tetratricopeptide repeat protein |
| NC_008095.1 | 6611869 | A | G | 2848.77 | missense | MXAN_RS38830 | hypothetical protein |
| NC_008095.1 | 7101832 | T | C | 3223.77 | synonymous | MXAN_RS27795 | signal recognition particle-docking protein FtsY |
| NC_008095.1 | 7244826 | C | A | 3797.77 | missense | MXAN_RS28305 | prephenate dehydrogenase/arogenate dehydrogenase family protein |
| NC_008095.1 | 7259425 | T | G | 4517.77 | missense | MXAN_RS28370 | ATP-binding protein |
| NC_008095.1 | 7460035 | C | A | 3703.77 | missense | MXAN_RS29245 | chemotaxis protein CheB |
| NC_008095.1 | 7583588 | G | T | 3383.77 | intergenic | - |  |
| NC_008095.1 | 8038368 | G | A | 3870.77 | missense | MXAN_RS31585 | methionine adenosyltransferase |
| NC_008095.1 | 8179131 | C | T | 4027.77 | synonymous | MXAN_RS32190 | hypothetical protein |
| NC_008095.1 | 8263368 | T | C | 4467.77 | intergenic | - |  |
| NC_008095.1 | 8450865 | G | A | 4147.77 | missense | MXAN_RS33405 | Ig-like domain-containing protein |
| NC_008095.1 | 8909897 | A | G | 4502.77 | missense | MXAN_RS35290 | carbohydrate ABC transporter permease |
| NC_008095.1 | 9093391 | G | C | 179.77 | intergenic | - |  |
| **InDels:** |  |  |  |  |  |  |  |
| Chromosome | Position | Reference | Alteration | Quality |  | Locus | Annotation |
| NC_008095.1 | 251058 | G | GT | 2675.73 |  | intergenic | - |
| NC_008095.1 | 5321929 | G | GC | 2065.73 |  | MXAN_RS20980 | AHH domain-containing protein |
| NC_008095.1 | 5999391 | AAT | A | 213.73 |  | MXAN_RS23320 | Ig-like domain-containing protein |
| NC_008095.1 | 5999394 | C | CGA | 213.73 |  | MXAN_RS23320 | Ig-like domain-containing protein |
| NC_008095.1 | 5999397 | ACCT | A | 177.73 |  | MXAN_RS23320 | Ig-like domain-containing protein |
| NC_008095.1 | 5999404 | G | GGCT | 183.73 |  | MXAN_RS23320 | Ig-like domain-containing protein |
| NC_008095.1 | 6070866 | C | CT | 548.73 |  | MXAN_RS23570 | IS3-like element ISMxa1 family transposase |
| NC_008095.1 | 8235891 | C | CCGCCAGCCCCATCAAGACGTAGCCGTTGAGAATCTTCCGGTGAAGGGAGACCTCGCGGGTCGCGCGAGG | 2120.73 |  | MXAN_RS32415 | MmcQ/YjbR family DNA-binding protein |
| NC_008095.1 | 8992406 | G | GC | 426.73 |  | intergenic | - |
| NC_008095.1 | 9093379 | CAG | C | 346.73 |  | intergenic | - |
| NC_008095.1 | 9093381 | G | GCCCCCCCCC | 355.73 |  | intergenic | - |
| **SV deletions:** |  |  |  |  |  |  |  |
| Chromosome | Position1 | Position2 | Type | Size |  | Locus | Annotation |
| NC_008095.1 | 161773 | 162101 | DEL | 316 |  | MXAN_RS00680 | GMC family oxidoreductase |
| NC_008095.1 | 187469 | 187878 | DEL | 404 |  | MXAN_RS00745 | methyltransferase domain-containing protein |
| NC_008095.1 | 1015931 | 1031123 | DEL | 15291 |  | BGC1 | Carotenoids synthesis cluster containing |
| NC_008095.1 | 1901962 | 1902264 | DEL | 319 |  | MXAN_RS07815 | non-ribosomal peptide synthetase |
| NC_008095.1 | 1962661 | 1963028 | DEL | 368 |  | MXAN_RS08055 | mazF |
| NC_008095.1 | 3505166 | 3507753 | DEL | 2700 |  | MXAN_RS14485 | aglZ |
| NC_008095.1 | 4943910 | 4944242 | DEL | 322 |  | MXAN_RS19615/MXAN_RS19620 | transglycosylase SLT domain-containing protein/glutathione S-transferase family protein |
| NC_008095.1 | 5235971 | 5311073 | DEL | 75190 |  | BGC17 | Dkxanthene synthesis cluster containing |
| NC_008095.1 | 5773414 | 5773763 | DEL | 352 |  | MXAN_RS22330 | non-ribosomal peptide synthetase |
| NC_008095.1 | 7158920 | 7159412 | DEL | 581 |  | MXAN_RS28035 | pilA |
| NC_008095.1 | 7182904 | 7183287 | DEL | 357 |  | MXAN_RS28115 | RHS repeat-associated core domain-containing protein |
| NC_008095.1 | 8235966 | 8237122 | DEL | 1253 |  | MXAN_RS32420 | difA |

SNP: single nucleotide polymorphism, InDel: insert and deletion, SV: structure variation

**Supplementary Table S3.** Bacterial strains and plasmids used in this study.

| Strains | Description | Source |
| --- | --- | --- |
| *M. xanthus* DK1622 | Wild-type strain | D. Kaiser (University of Standford) |
| *M. xanthus* WY01 | ΔaglZ | This study |
| *M. xanthus* WY02 | ΔaglZ; ΔpilA; | This study |
| *M. xanthus* WY03 | ΔaglZ; ΔpilA; ΔdifA | This study |
| *M. xanthus* Hu02 | ΔaglZ; ΔpilA; ΔdifA; ΔBGC17 | This study |
| *M. xanthus* Hu03 | ΔaglZ; ΔpilA; ΔdifA; ΔBGC17; ΔBGC1 | This study |
| *M. xanthus* Hu04 | ΔaglZ; ΔpilA; ΔdifA; ΔBGC17; ΔBGC1; ΔmazF | This study |
| Hu04-eGFP | Expression of fluorescent protein eGFP | This study |
| Hu04-MchA | Expression of *mchA* gene | This study |
| *E. coli* Top10 | F^-^ mcrA Δ(mrr-hsdRMS-mcrBC) Φ80 lacZΔM15 ΔlacX74 recA1 araΔ139 Δ(ara,-leu)7697 galU galK rpsL(Str^R^) endA1 nupG | Laboratory collection |
|  |  |  |
| Plasmids | Characteristics | Source |
| pBJ113 | ColE1 ori, *kan*^R^, *galK*, f1 ori, LacZα-peptide, MCS | Laboratory collection |
| pZJY41 | ColE1 ori, PMF1 ori, *kan*^R^ | Laboratory collection |
| pBJ113-*ΔpilA* | pBJ113, ~1 Kb homologous arms before and after *pilA* | This study |
| pBJ113-*ΔdifA* | pBJ113, ~1 Kb homologous arms before and after *difA* | This study |
| pBJ113-*ΔaglZ* | pBJ113, ~1 Kb homologous arms before and after *aglZ* | This study |
| pBJ113-*Δ*BGC17 | pBJ113, ~1 Kb homologous arms before and after the predicted region of BGC17 | This study |
| pBJ113-*Δ*BGC1 | pBJ113, ~1 Kb homologous arms before and after the predicted region of BGC1 | This study |
| pBJ113-*ΔmazF* | pBJ113, ~1 Kb homologous arms before and after *mazF* | This study |
| pZJY41-eGFP | pZJY41, pSH058-B0034-eGFP-B0015 | This study |
| pBJ113-eGFP | pBJ113, eGFP | This study |
| pBJ113-Lachis | pBJ113, *LacI*, BBa_J23104-LacO-B0034-N terminal 6×His | This study |
| pTim-S0 | ColE1 ori, PMF1 ori, *apra*^R^ | This study |
| pHu-*mchA* | ColE1 ori, PMF1 ori, *apra*^R^, *LacI*, BBa_J23104-LacO-B0034-N terminal 6×His of *MchA* | This study |

**Supplementary Table S4.** Primers used in this study.

| Primers | Primer sequences (5'-3') | | Application |
| --- | --- | --- | --- |
| 113-F | | GAATTCACTGGCCGTCGTTT | pBJ113 linearization |
| 113-R | | TCTAGAGTCGACCTGCAGGC |  |
| aglZ-UF | | AAACGACGGCCAGTGAATTCGGAGCTCATCCCCACGCG | *aglZ* homologous arms |
| aglZ-UD | | TGCGCCTTGATGGCGTCGCGATCGCGAGGACGGTGTCCTGGAGC |  |
| aglZ-DF | | TCGCGACGCCATCAAGGCGCA |  |
| aglZ-DR | | GCCTGCAGGTCGACTCTAGAGCTGGGCCTTCAGCACCGGGC |  |
| pilA-UF | | AAACGACGGCCAGTGAATTCAACTGCGCGGCGTTGAAC | *pilA* homologous arms |
| pilA-UD | | CGCGTGTTGTAGGGGGTACCGCGATGAGCGTGAAGCCACGG |  |
| pilA-DF | | CGGTACCCCCTACAACACGCG |  |
| pilA-DR | | GCCTGCAGGTCGACTCTAGACTCCTCCGGAAGGTCGAAC |  |
| difA-UF | | AAACGACGGCCAGTGAATTCGTTCGATTTCATCCGCCGGT | *difA* homologous arms |
| difA-UD | | TTGTCCGAGGGCGTCTGGCCGCCAGCCCCATCAAGACG |  |
| difA-DF | | GCCAGACGCCCTCGGACAA |  |
| difA-DR | | GCCTGCAGGTCGACTCTAGAAGTAGAACCACCCGTCCGGG |  |
| BGC17-UF | | AAACGACGGCCAGTGAATTCGCAGCGTCTTGGAATCCTCC | BGC17 homologous arms |
| BGC17-UD | | TGGAGGTGTCGTTCGACCGGAGCACCGTCGACAACTCAC |  |
| BGC17-DF | | CGGTCGAACGACACCTCCA |  |
| BGC17-DR | | GCCTGCAGGTCGACTCTAGATTCGTCATCGGCTTCCTCGC |  |
| BGC1-UF | | AAACGACGGCCAGTGAATTCGTGGGGCGACAACACGTTCGGCCAA | BGC1 homologous arms |
| BGC1-UD | | CTCTGTCACCGCGTACTCCAACAGGCCCTGCTGCCCGG |  |
| BGC1-DF | | TGGAGTACGCGGTGACAGAGCATCC |  |
| BGC1-DR | | GCCTGCAGGTCGACTCTAGAGCTTCGTGCACATCCCCG |  |
| mazF-UF | | AAACGACGGCCAGTGAATTCCCGGTGGCCACGTCCCAGAT | *mazF* homologous arms |
| mazF-UD | | GCCCACTCACCGGGGCTTGCCCTCGGGGTCTCCTGT |  |
| mazF-DF | | AAGCCCCGGTGAGTGGGC |  |
| mazF-DR | | GCCTGCAGGTCGACTCTAGATCGAATGGCACGCGCGGC |  |
| pZJY41-F | | GAATTCTTTTTTCCAGGCATCAAATAAAACGAAAG | pZJY41 linearization |
| pZJY41-R | | TCTTCCGCTTCCTCGCTCACT |  |
| eGFP-F(058) | | GTGAGCGAGGAAGCGGAAGATTGACACAGCCCAGCCGG | eGFP amplification |
| eGFP-R | | TGCCTGGAAAAAAGAATTCTCACTTGTACAGCTCATCCATGCC |  |
| mchA-F | | CTGCAGTTCAGAACGCTCGG | *mchA* amplification |
| mchA-R | | ATCCTCTAGAGGATCCCCAATCATGCCGTCTCCCTCTCCT |  |
| lacI-F | | CGATGCGAGGAAACGACGATGGACATGGTGAAGCCGGT | *lacI* amplification |
| lacI-R | | CAAAAAACCCCTCAAGACCCGTT |  |
| Apra/Pvan-F | | CGTCGTTTCCTCGCATCG | Apra/Pvan fragment amplification |
| Apra/Pvan-R | | CCGAGCGTTCTGAACTGCAGTCAGCCAATCGACTGGCGAG |  |
| ColE/PMF1-F | | TTGGGGATCCTCTAGAGGATTCTTCCGCTTCCTCGCTCACT | ColE/ PMF1 ori fragment amplification |
| ColE/PMF1-R | | GGGTCTTGAGGGGTTTTTTGCTGAAAGGAGGAACTATATCCGGATCAT |  |

**Supplementary Figure S1.** Gene deletions and mutations in *M. xanthus* Hu04. **(A)** Distribution of variations across the Hu04 genome. From outer to inner: deleted genes and regions to be deleted; chromosome (light blue); SNP (single nucleotide polymorphism) density; InDel (insert and deletion) density; SV (structure variation) deletion; CNV (copy number variation) deletion; ITX (intra-chromosomal translocation). **(B)** Information about the deleted genes. Genes in the BGC1 and BGC17 were documented in Supplementary Table S1.


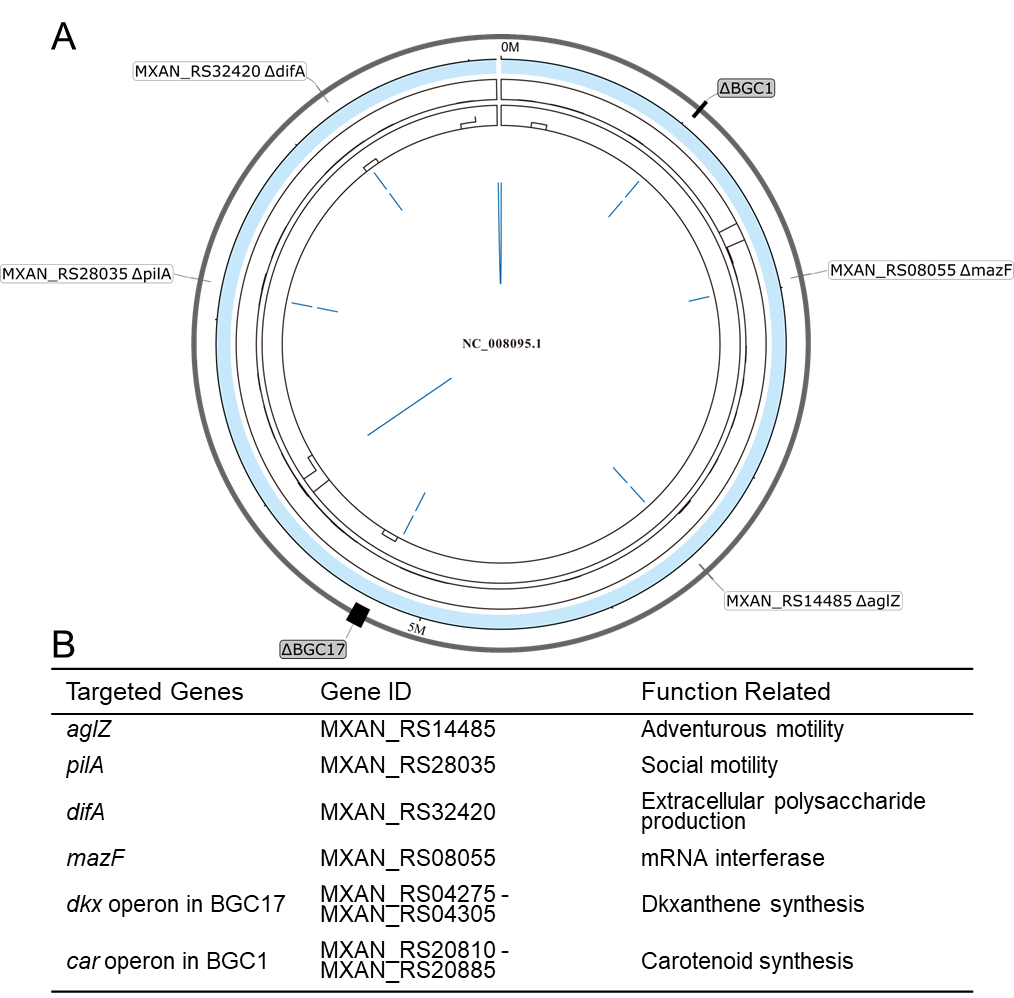


**Supplementary Figure S2.** Purification of the MchA protein in Hu04. **(A)** The color change in Hu04-MchA resulting from the induction of MchA by IPTG. **(B)** SDS-PAGE analysis of the His-MchA purified from Hu04-MchA. The imidazole concentrations in the elution buffer were 20, 50, 75, 100, and 125 mM. "-" denotes the control group without the addition of the inducer IPTG, while "+" represents the group with the inducer added. Due to expression leakage, a small amount of MchA protein was also purified in the uninduced control group.


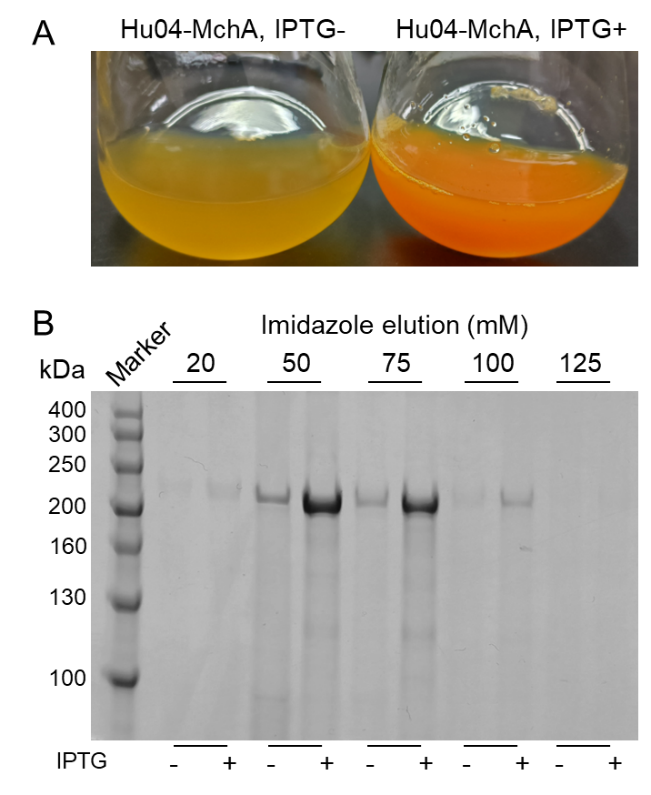


**References：**

[1] W.-F. Hu, L. Niu, X.-J. Yue, L.-L. Zhu, W. Hu, Y.-Z. Li, C. Wu, Characterization of Constitutive Promoters for the Elicitation of Secondary Metabolites in Myxobacteria., ACS Synth Biol 10 (2021) 2904–2909. https://doi.org/10.1021/acssynbio.1c00444.

[2] L.-P. Zhu, X.-J. Yue, K. Han, Z.-F. Li, L.-S. Zheng, X.-N. Yi, H.-L. Wang, Y.-M. Zhang, Y.-Z. Li, Allopatric integrations selectively change host transcriptomes, leading to varied expression efficiencies of exotic genes in Myxococcus xanthus., Microb Cell Fact 14 (2015) 105. https://doi.org/10.1186/s12934-015-0294-5.

[3] W. Shi, D.R. Zusman, The two motility systems of Myxococcus xanthus show different selective advantages on various surfaces., P Natl Acad Sci Usa 90 (1993) 3378–3382. https://doi.org/10.1073/pnas.90.8.3378.
